# Cervico-vaginal microbiota and antimicrobial resistance patterns in relation to different reproductive statuses in cattle: a field study from Nepal

**DOI:** 10.3389/frmbi.2026.1900716

**Published:** 2026-10-05

**Authors:** Bishal Bhattachan, Gokarna Gautam, Rabin Raut, Hom Bahadur Basnet, Bibek Bhattachan, Sundar Khatiwada, Rebanta Kumar Bhattarai

**Affiliations:** 1 Department of Veterinary Microbiology and Parasitology, Agriculture and Forestry University, Bharatpur, Nepal; 2 National Veterinary Medicine Center, Bharatpur, Nepal

**Keywords:** 16S rRNA gene sequencing, antimicrobial resistance, Bovine vaginal microbiota, Multidrug-resistant bacteria, reproductive status in cattle

## Abstract

Vaginal bacterial communities are increasingly recognized as key determinants of fertility in cattle. This study explored bacterial communities in the cervico-vaginal mucus of healthy heifers, cows with cervicitis, repeat-breeding, and cows within the 35-day postpartum period using the microbiological culture method (n=35) and sequencing of the V3-V4 hypervariable regions of 16S rRNA gene (n=18), along with antimicrobial susceptibility testing. *Micrococcus* spp. and *Enterobacter* spp. were the top two bacteria isolated across the groups. Alarmingly, antimicrobial sensitivity tests revealed a high prevalence of multidrug resistance (71.7%) and elevated multiple antibiotic resistance indices in the isolated bacteria; none of the isolates were sensitive to all the tested bacteria. From 16S rRNA sequencing, a total of 1,047,482 sequences and 2,366 operational taxonomic units (OTUs) were generated. All groups shared 434 OTUs, and they did not differ in alpha diversity and beta diversity except for healthy heifers and cervicitis cattle. Bacillota, followed by Pseudomonadota, Fusobacteriota, Actinomycetota, Bacteroidota, and Mycoplasmatota dominated the vaginal microbiota across all groups. Genus-level comparisons revealed the following group-specific enriched taxa: *Chryseobacterium* with a linear discriminant analysis (LDA) score > 3 in cervicitis, *Filifactor* (LDA score > 2) in repeat breeding cow, and *Histophilus* (LDA score > 3.5) in healthy heifers. These findings offer important insights into the structure of bovine vaginal microbial communities while highlighting the urgent need to monitor antimicrobial resistance. This study lays the groundwork for defining the core vaginal microbiota and its potential implications for reproductive health and disease. To the best of our knowledge, this is the first study in Nepal to investigate cervico-vaginal microbiota and antimicrobial resistance patterns in cattle across different reproductive statuses using both culture-dependent methods and 16S rRNA gene sequencing.

## 1 Introduction

Reproductive efficiency is a critical determinant of sustainability and profitability in the dairy industry; however, reproductive failure continues to impose a substantial economic burden, with infertility in dairy cattle accounting for an estimated USD 12.6 billion annual global loss (Rasmussen et al., 2024). Major contributing conditions include repeat breeding, postpartum anestrus, and cervicitis, which collectively prolong the calving interval and reduce lifetime productivity (Peter et al., 2009; Perez‐Arnedo et al., 2021). These disorders are closely associated with the postpartum period, during which uterine involution, immune suppression, and microbial invasion increase susceptibility to infections such as metritis, endometritis, and cervicitis (Carvalho et al., 2014; Banuelos and Stevenson, 2021). Among them, bacterial infections of the reproductive tract are considered a major underlying factor, while the increasing prevalence of antimicrobial resistance (AMR) further complicates treatment and control strategies (Sheldon et al., 2006).

The reproductive microbiota plays a fundamental role in maintaining uterine and vaginal health, fertility, and pregnancy outcomes. In cattle, the vaginal microbiota contributes to the uterine microbial environment through ascension during estrus, breeding, and parturition, particularly when the cervical barrier is compromised (Galvão et al., 2019; Moreno et al., 2022). The cervix acts as a key anatomical and immunological barrier, supported by mucus-mediated antimicrobial protection (Azawi, 2008). Disruption of microbial balance (dysbiosis), characterized by reduced diversity and overgrowth of opportunistic pathogens such as *Escherichia coli*, *Trueperella pyogenes*, and *Fusobacterium* spp., has been associated with reproductive disorders and reduced fertility (Galvão et al., 2019; Pascottini et al., 2020). The bovine reproductive tract microbiota is dominated by phyla including Bacillota, Mycoplasmatota, Actinomycetota, Bacteroidota, Proteobacteriota, and Fusobacteriota. These groups include both beneficial and pathogenic taxa, such as spore-forming Firmicutes (*Clostridium*, *Bacillus*), cell wall–deficient Mycoplasmatota (*Mycoplasma*, *Ureaplasma*), and opportunistic pathogens within Proteobacteriota and Fusobacteriota. These microbial communities collectively influence reproductive tract homeostasis, pathogen resistance, and host immune responses (Bronzo et al., 2020).

Despite its importance, the bovine reproductive microbiome remains poorly characterized compared to humans, where *Lactobacillus*-dominated communities are strongly associated with reproductive health (Swartz et al., 2014). In cattle, however, limited baseline data exist regarding vaginal microbial composition across different reproductive statuses, particularly in relation to fertility disorders. Moreover, although culture-based methods have been widely used, they capture only a fraction of microbial diversity due to the presence of unculturable organisms (Clemmons et al., 2017). In contrast, 16S rRNA gene sequencing enables comprehensive microbial profiling, with the V3–V4 region providing high-resolution identification of bacterial taxa (Yang et al., 2016; Graspeuntner et al., 2018).

In Nepal, reproductive disorders such as repeat breeding and anestrus are highly prevalent, ranging from 0.5–31% and 24%, respectively, contributing significantly to economic losses in dairy production systems (Pun et al., 2024; Sharma et al., 2025). Given the role of bacterial infections and rising antimicrobial resistance, there is an urgent need to characterize the reproductive microbiota and its resistance patterns to improve diagnostic and therapeutic strategies. Therefore, this study aimed to characterize the cervico-vaginal microbiota and antimicrobial resistance patterns in cattle with different reproductive statuses using both culture-based methods and 16S rRNA gene sequencing, to provide baseline data for understanding microbial associations with reproductive health and disease.

## 2 Materials and methods

### 2.1 Ethics statement

Ethical clearance for this study was obtained from the Institutional Review Board of Agriculture and Forestry University (AFU), Nepal.

### 2.2 Study design and animal selection

The study was conducted from July 2024 to April 2025 in Sainamaina Municipality, Rupandehi District (27.6934° N, 83.3148° E) and Bharatpur Metropolitan City, Chitwan (27.6706° N, 84.4385° E), including the AFU livestock farm and the National Cattle Research Program (NCRP). A cross-sectional study design with convenience sampling was employed. The study included healthy heifers, cows with cervicitis, repeat breeders, cows within 35 days postpartum, and postpartum anestrus cows. Animals treated with antibiotics or having non-reproductive infections were excluded.

Healthy heifers were defined as females under two years of age with a body condition score of 3–3.5 and no clinical signs of disease. Since the vaginal microbiome of heifers is not influenced by assisted reproductive technologies or pregnancy, it reflects the natural vaginal microbiome (Moreno et al., 2021). Cervicitis cases were identified as cows with an enlarged cervix, especially the posterior end at rectal palpation, and severe congestion and protrusion (eversion) of the external cervical os at the vaginoscopy examination. Repeat breeders were defined as cows with normal estrous cycles but failure to conceive after three or more inseminations without other reproductive abnormalities (based on history). Postpartum cows within 35 days were included based on normal calving history. Postpartum anestrus cows were defined as those not exhibiting estrus after 60 days postpartum and were further classified by ultrasonography as silent heat (presence of corpus luteum) or true anestrus (absence of corpus luteum).

### 2.3 Sample collection

Vaginal and cervical samples were collected using a sterile cytobrush under proper animal restraint. The perineal area was cleaned with water, disinfected with povidone-iodine, and dried prior to sampling. A sterilized vaginoscope was used to visualize the external os of the cervix (sample collection site) and cranial vagina (sample collection site), and the cytobrush (covered with a sterile AI sheath) was carefully introduced without contact with vaginal walls. The cytobrush was rotated for approximately 30 seconds to collect cervical and vaginal mucus. Immediately after collection, samples were transferred into sterile tubes containing phosphate-buffered saline (PBS, pH 7.0) for vaginal samples and brain heart infusion (BHI) broth for cervical samples. Samples were stored on ice during transport, then kept at 4 °C and subsequently preserved at −80 °C until further microbiological and molecular analysis in the Veterinary Microbiology Laboratory, AFU, Nepal.

A total of 35 samples were initially collected from Sainamaina Municipality for culture and sequencing (**Table 1**). However, during DNA extraction, some samples from Sainamaina presented compromised DNA concentrations and purities. To ensure a minimum of three samples for each case type, additional samples were collected from the AFU and NCRP for 16S rRNA sequencing (**Table 2**).

**Table 1:** Total number of samples collected from different cases of cattle.

Groups	Number of animals
True anestrus	17
Silent heat	6
Repeat breeding (RB)	4
Cows within the 35-day postpartum period (PP)	4
Cervicitis (C)	2
Healthy heifers (Hf)	2
Total	35

**Table 2:** Total number of samples used for 16S rRNA sequencing with their source.

Groups	Source (frequency)	Total
Healthy heifers (Hf)	NCRP (3), Sainamaina (2)	5
Cervicitis (C)	AFU (4), Sainamaina (1)	5
Repeat breeding (RB)	Sainamaina (3)	3
Cows within the 35-day postpartum period (PP)	NCRP (2), Sainamaina (3)	5
Total		18

Value within parentheses represents frequency.

### 2.4 Isolation and identification of bacteria

Each sample was transferred onto Nutrient agar (M001, HiMedia, India), Blood agar (M073, HiMedia, India), and MacConkey agar (M081A, HiMedia, India) plates by streaking with a sterile inoculation loop. Then, the inoculated agar plates were incubated aerobically at 37 °C for 24 h. After incubation at 37 °C for 24 to 48 h, bacteria were identified based on their colonial morphology, Gram’s staining, hemolysis, and biochemical tests (Bauer et al., 1966).

### 2.5 Antimicrobial susceptibility testing

Antimicrobial susceptibility testing of bacterial isolates was performed using the Kirby–Bauer disk diffusion method (Bauer et al., 1966). A panel of antibiotics, including tetracycline (30 µg), ampicillin (10 µg), sulfamethoxazole (25 µg), ciprofloxacin (5 µg), chloramphenicol (30 µg), erythromycin (15 µg), ceftriaxone (30 µg), and gentamicin (10 µg) (HiMedia, India) was used. Fresh bacterial colonies were inoculated into nutrient broth and incubated at 37 °C for 5 h until turbidity developed, which was adjusted to 0.5 McFarland standard. A standardized inoculum (100 µl) was spread onto Mueller–Hinton agar plates, and antibiotic disks were applied aseptically. Plates were incubated aerobically at 37 °C for 24 h, and zones of inhibition were measured and interpreted as sensitive, intermediate, or resistant according to CLSI guidelines (CLSI, 2023). For antimicrobial resistance analysis, only bacterial species with sufficient number of isolates were included to enable meaningful interpretation. Species represented by three or less than three isolates were excluded from antimicrobial analysis because the limited size did not permit reliable estimation of resistance patterns.

Multidrug resistance (MDR) was determined based on the antimicrobial susceptibility results, defined as resistance to at least one agent in three or more antibiotic classes, according to Magiorakos et al. (2012). The multiple antibiotic resistance (MAR) index (a/b) was calculated for each isolate based on its resistance profile as the ratio of the number of antibiotics to which an isolate was resistant (i.e., “a” herein) to the total number of antibiotics tested (i.e., “b” herein) (Krumperman, 1983). The isolates with MAR index > 0.2 are considered to come from high-risk sources where antibiotics are frequently used or misused. Similarly, the isolates with MAR index ≤ 0.2 are interpreted as originating from low-risk sources with limited antibiotic exposure.

### 2.6 DNA extraction and quantification

Bacterial DNA was extracted from cervico-vaginal mucus samples using the DNeasy® Blood & Tissue kit (catalog number 69504, Qiagen, Hilden, Germany) following the manufacturer's protocol with minor modifications (Druker et al., 2024). Briefly, samples were thawed at room temperature and lysed using AL buffer, ATL buffer, and proteinase K, followed by incubation at 56 °C for 1 h. For mucus-rich samples, the proteinase K volume was increased. DNA purification and elution were performed according to the manufacturer’s instructions, and purified DNA was stored in elution buffer at –80 °C.

DNA concentration and purity were assessed using a spectrophotometer (Thermo Fisher Scientific, USA) at 260/280 nm, with purity ratios of 1.7–1.9 considered acceptable. DNA integrity was confirmed by electrophoresis on a 1% agarose gel, and high-quality DNA samples were stored at –20 °C for downstream analysis. The extracted DNA was sent to BGI genomics Hongkong for sequencing.

### 2.7 Bioinformatics analysis of microbiome data

PCR and other downstream workflow were done using BGI genomics, Hongkong standard protocol. Briefly, total genomic DNA (30 ng per sample) was used for PCR amplification of the V3–V4 region of the bacterial 16S rRNA gene using primers 341F (5′- CCTACGGGNGGCWGCAG-3′) and 806R (5′-GGACTACHVGGGTWTCTAAT-3′). PCR products were purified using Agencourt AMPure XP beads (Beckman Coulter, USA), and library quality was assessed using an Agilent 2100 Bioanalyzer (Agilent Technologies, USA). Qualified libraries were sequenced on a DNA nanoball (DNB) sequencing platform. Raw reads were processed to obtain high-quality clean sequences by trimming low-quality bases (Phred score <20 over a 25 bp sliding window), removing adapter-contaminated reads using Cutadapt (v2.6), and filtering reads containing ambiguous bases or low complexity. Paired-end reads were merged using FLASH (v1.2.11) with a minimum overlap of 15 bp and a maximum mismatch ratio of 0.1. High-quality sequences were clustered into operational taxonomic units (OTUs) at 97% similarity using USEARCH (v7.0.1090) with the UPARSE algorithm, and chimeric sequences were removed using UCHIME (v4.2.40). Taxonomic assignment was performed using the RDP classifier (v2.2) against the Greengenes2 (version V202210).

Alpha diversity indices (Shannon, Simpson, and Chao1) were calculated using Mothur (v1.31.2) to assess within-sample diversity, while beta diversity was evaluated using Bray–Curtis dissimilarity and visualized through principal coordinates analysis (PCoA). Differentially abundant taxa among groups were identified using Linear Discriminant Analysis Effect Size (LEfSe) with an LDA score threshold of 2.0.

## 3 Statistical analysis

Differences in alpha diversity and taxon abundance among groups were assessed using the Kruskal–Wallis test. Beta diversity differences were evaluated using permutational multivariate analysis of variance (PERMANOVA/Adonis). Pairwise comparisons were performed using the Wilcoxon test. Spearman correlation analysis was used to assess relationships among bacterial taxa. Statistical significance was set at P < 0.05. All analyses were conducted in R software (v3.1.1; www.R-project.org).

## 4 Results

### 4.1 Distribution of bacterial growth in vaginal samples across different reproductive categories of cattle

Out of the 35 samples collected from different categories of cattle, bacterial growth was detected in 30 (85.7%) samples. Notably, bacterial growth was detected in all samples from true anestrous cows, cows within the 35-day postpartum period, and cows with cervicitis (**Table 3**).

**Table 3:** Distribution of bacterial growth from vaginal samples across different reproductive categories of cattle.

Groups	Growth	No growth	Total
True anestrus	17 (48.5)	0	17 (48.5)
Silent heat	3 (8.6)	3 (8.6)	6 (17.1)
Repeat breeding	3 (8.6)	1 (2.8)	4 (11.4)
Cows within the 35-day postpartum period	4 (11.4)	0	4 (11.4)
Cervicitis	2 (5.7)	0	2 (5.7)
Healthy heifers	1 (2.8)	1 (2.8)	2 (5.7)
Total	30 (85.7)	5 (14.3)	35

Values within the parentheses represent percentages.

### 4.2 Identification of bacteria

Out of the total 54 bacterial isolates, 55.5% (30 isolates) were Gram-negative, while the remaining 44.5% (24 isolates) were Gram-positive. Among the Gram-negative isolates, members of the Enterobacteriaceae family predominated, *Enterobacter* spp. (20.3%, 11 isolates), *Acinetobacter* spp. (13.0%, 7 isolates), *Serratia* spp. (5.5%, 3 isolates), *Salmonella* spp. (9.2%, 5 isolates), *Pseudomonas* spp. (1.8%, 1 isolate), *Shigella* spp. (1.8%, 1 isolate), *Citrobacter* spp. (1.8%, 1 isolate), and *E. coli* (1.8%, 1 isolate), accounting for 55.5% (30 isolates). Among the Gram-positive isolates, *Micrococcus* spp. (22.2%, 12 isolates) was the most prevalent, followed by *Staphylococcus* spp. (11.1%, 6 isolates), *Bacillus* spp. (9.2%, 5 isolates), and *Corynebacterium* spp. (1.8%, 1 isolate) (**Table 4**).

**Table 4:** Bacterial isolates from cows with different reproductive conditions.

Bacteria	PP	True anestrus	Cervicitis	Repeat breeding	Silent heat	Total
*Staphylococcus* spp.	1	3	0	2	0	6
*Micrococcus* spp.	2	9	1	0	0	12
*Enterobacter* spp.	0	8	1	0	2	11
*Bacillus* spp.	1	3	0	0	1	5
*Acinetobacter* spp.	0	2	1	2	2	7
*Serratia* spp.	0	2	0	1	0	3
*Salmonella* spp.	1	4	0	0	0	5
*Shigella* spp.	0	1	0	0	0	1
*Pseudomonas* spp.	1	0	0	0	0	1
*Citrobacter* spp.	0	1	0	0	0	1
*E. coli*	1	0	0	0	0	1
*Corynebacterium* spp.	0	0	0	0	1	1
Total	7	33	3	5	6	54

PP, Cows within the 35-day postpartum period.

### 4.3 Antibiotic susceptibility pattern of bacteria to antibiotics

*Staphylococcus* spp. showed the highest susceptibility to chloramphenicol (100%), followed by gentamicin and ciprofloxacin (83.3% each), but were completely resistant to ampicillin (100%) and highly resistant to erythromycin (83.3%) (**Table 5**). *Salmonella* spp. exhibited complete resistance to ciprofloxacin (100%) and high resistance to ampicillin (80%), while remaining susceptible to gentamicin (80%). *Bacillus* spp. were fully resistant to ampicillin (100%) but showed moderate susceptibility to gentamicin, sulfamethoxazole, and chloramphenicol (80% each). *Acinetobacter* spp. showed high resistance to ciprofloxacin (85.7%) and ampicillin (71.4%), while remaining susceptible to gentamicin and tetracycline (71.4% each). *Micrococcus* spp. were highly resistant to ceftriaxone and ampicillin (100%), as well as erythromycin (83.3%), but remained susceptible to chloramphenicol (100%), gentamicin (83.3%), and ciprofloxacin (75%). *Enterobacter* spp. showed resistance to ampicillin and ciprofloxacin (72.7%), while being fully susceptible to gentamicin (100%) and highly susceptible to tetracycline (90.9%).

**Table 5:** Antibiotic susceptibility pattern of bacteria to antibiotics.

Bacteria		GM	CRO	AP	E	CIP	T	TS	C	Total
*Staphylococcus* spp.	R	1	2	6	5	0	3	2	0	19
	I	0	2	0	1	1	1	0	0	5
	S	5	2	0	0	5	2	4	6	24
*Salmonella* spp.	R	1	3	4	0	5	2	1	1	17
	I	0	1	0	5	0	0	1	1	8
	S	4	1	1	0	0	3	3	3	15
*Micrococcus* spp.	R	2	12	12	10	0	3	2	0	41
	I	0	0	0	1	3	0	2	0	6
	S	10	0	0	1	9	9	8	12	49
*Enterobacter* spp.	R	0	6	8	7	8	1	1	1	32
	I	0	1	2	4	1	0	4	4	16
	S	11	4	1	0	2	10	6	6	40
*Bacillus* spp.	R	1	1	5	2	1	1	1	0	12
	I	0	3	0	2	2	2	0	1	10
	S	4	1	0	1	2	2	4	4	18
*Acinetobacter* spp.	R	0	2	5	3	6	1	2	1	20
	I	1	5	2	3	0	0	0	1	12
	S	6	0	0	1	1	6	5	5	24

GM, Gentamicin; CRO, Ceftriaxone; CIP, Ciprofloxacin; AP, Ampicillin; E, Erythromycin; T, Tetracycline; TS, Sulphamethoxazole; C, Chloramphenicol; R, Resistance; I, Intermediate; and S, Sensitive.

Overall, none of the isolates were sensitive to all tested antibiotics. MDR was observed in 71.7% of isolates (**Table 6**), and 91.3% exhibited a MAR index greater than 0.2 (**Table 7**).

**Table 6:** Multidrug resistance (MDR) of the isolated bacteria was tested with eight antibiotics from different groups.

Bacteria	Number of isolates resistant	Number of antibiotic groups resistant	MDR	Overall
*Salmonella* spp.	1 (20)	1	–	1 (20)
	2 (40)	3	+	4 (80)
	1 (20)	4	+	
	1 (20)	6	+	
*Staphylococcus* spp.	1 (16.7)	1	–	1 (16.7)
	2 (33.3)	3	+	5 (83.3)
	3 (50)	4	+	
*Micrococcus* spp.	3 (25)	2	–	3 (25)
	9 (75)	3	+	9 (75)
*Enterobacter* spp.	3 (27.3)	2	–	3 (27.3)
	3 (27.3)	3	+	8 (73.7)
	4 (36.4)	4	+	
	1 (9.1)	5	+	
*Acinetobacter* spp.	1(14.2)	1	–	2 (28.5)
	1 (14.2)	2	–	
	3 (42.8)	3	+	5 (71.4)
	1 (14.3)	4	+	
	1 (14.3)	6	+	
*Bacillus* spp.	1 (20)	1	–	3 (60)
	2 (40)	2	–	
	1 (20)	3	+	2 (40)
	1 (20)	5	+	

Value within the parentheses indicates percentage. MDR, Multidrug resistance.

**Table 7:** Multiple antibiotic resistance (MAR) indices of the isolated bacteria were tested with eight antibiotics from different groups.

Bacteria	Number of isolates resistant	Number of antibiotic groups resistant	MAR index
*Salmonella* spp.	1 (20)	1	0.1
	2 (40)	3	0.4
	1 (20)	4	0.5
	1 (20)	6	0.8
*Staphylococcus* spp.	1 (16.7)	1	0.1
	2 (33.3)	3	0.4
	3 (50)	4	0.5
*Micrococcus* spp.	3 (25)	2	0.3
	9 (75)	3	0.4
*Enterobacter* spp.	3 (27.3)	2	0.3
	3 (27.3)	3	0.4
	4 (36.4)	4	0.5
	1 (9.1)	5	0.6
*Acinetobacter* spp.	1(14.2)	1	0.1
	1 (14.2)	2	0.3
	3 (42.8)	3	0.4
	1 (14.3)	4	0.5
	1 (14.3)	6	0.8
*Bacillus* spp.	1 (20)	1	0.1
	2 (40)	2	0.3
	1 (20)	3	0.4
	1 (20)	5	0.6

Value within the parentheses indicates percentage. MAR = Multiple antibiotic resistance.

### 4.4 Sequencing output and OTU analysis

A total of 18 samples were sequenced, generating 1,047,482 high-quality reads with a data utilization rate exceeding 99.5% after filtering. The data utilization rate exceeded 99.5% across all samples, indicating minimal loss during quality-control processing. Pair-end read assembly was highly efficient with merge rates ranging from 95.08% to 97.73%, resulting in 60,353-66,842 high quality merged sequences per sample (Supplementary 1). Species accumulation curves approached saturation, indicating adequate sequencing depth to capture microbial diversity. At a 97% similarity threshold, 2,366 OTUs were identified. The OTU table was normalized by random rarefaction to the minimum sampling depth across all samples prior to diversity analysis (Supplementary 2). A total of 434 OTUs were shared among all groups, representing a core microbiota across reproductive statuses. Group-specific OTU analysis revealed the highest number of unique OTUs in healthy heifers (414), followed by cows within 35 days postpartum (230), cervicitis cases (143), and repeat-breeding cows (83) (**Figure 1**).

**Figure 1:**
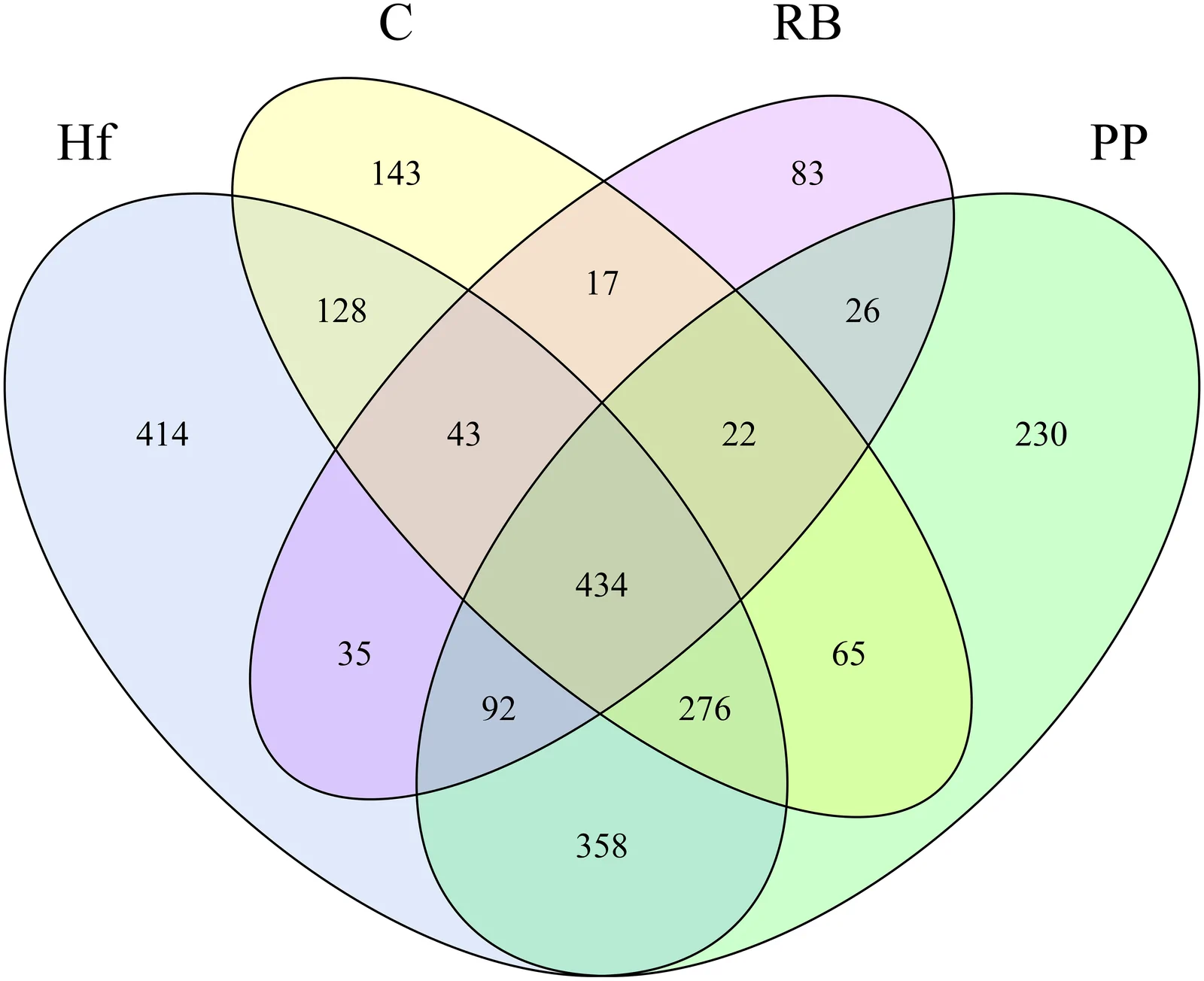
Operational taxonomic unit distribution across the groups. Hf, Healthy heifers; C, Cervicitis; RB, Repeat breeding; PP, Cows within 35 days postpartum period.

### 4.5 Alpha and beta diversity

Alpha diversity indices (Chao1, Shannon, and Simpson) showed no significant differences among the study groups (healthy heifers, cervicitis, postpartum, and repeat breeding cows) (**Figure 2**). However, pairwise comparison revealed significantly higher OTU richness in healthy heifers compared to cows with cervicitis (**Figure 3**).

**Figure 2:**
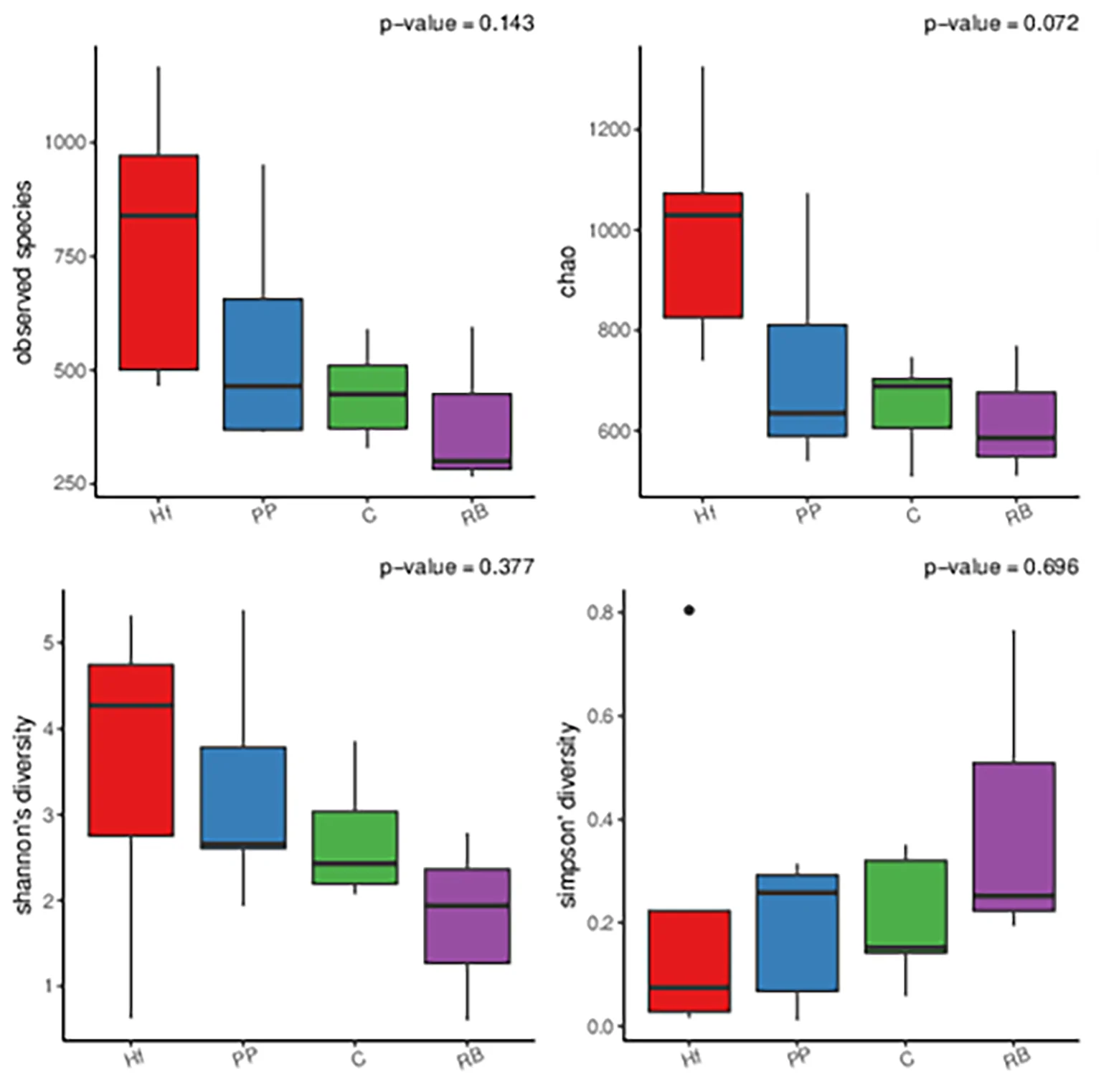
Boxplot of comparison of alpha diversity across the groups. Hf, Healthy heifers; C, Cervicitis; PP, Cows within 35 days postpartum; RB, Repeat breeding.

**Figure 3:**
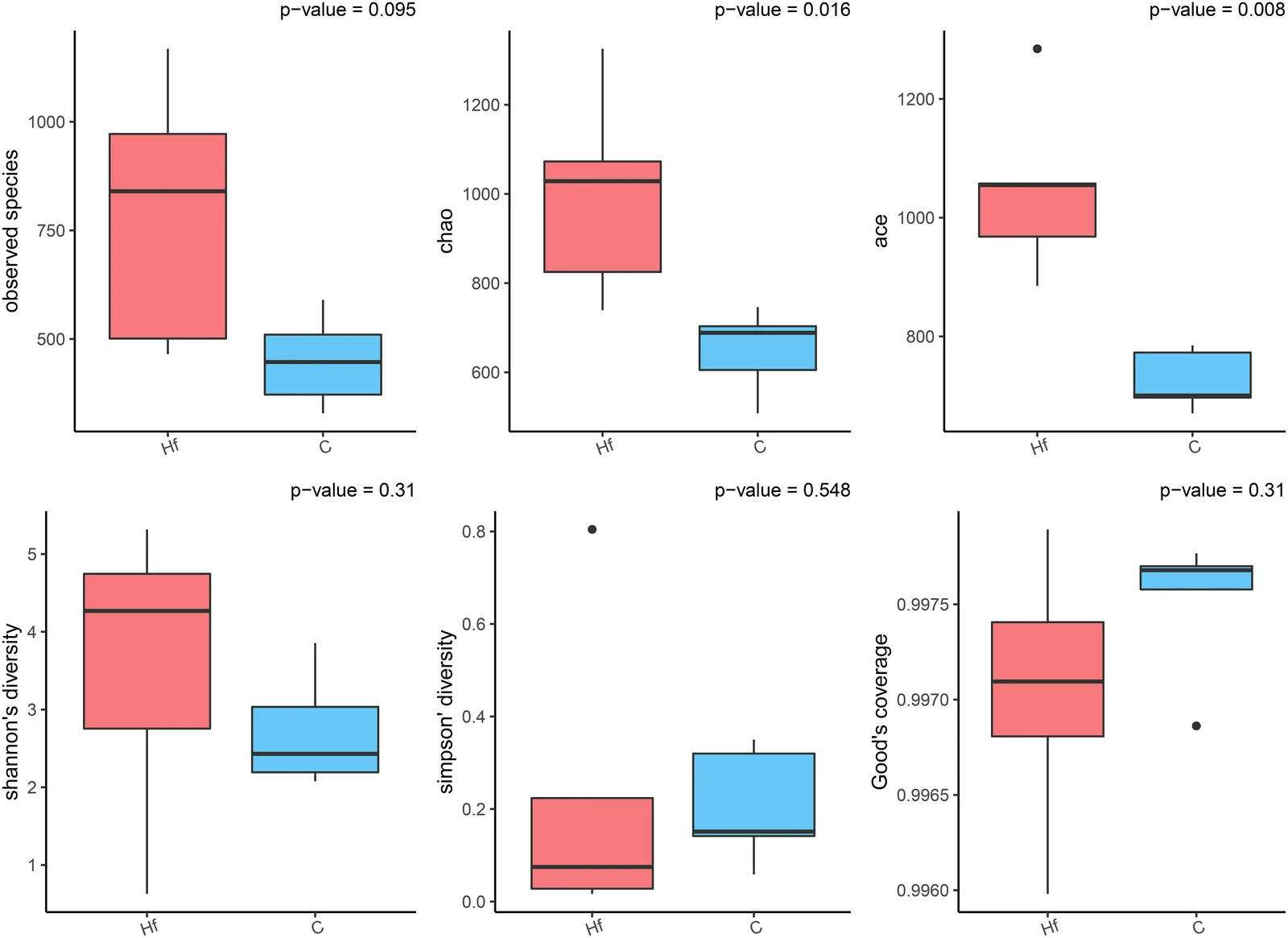
Boxplot of comparison of alpha diversity between healthy heifers (HF) and cervicitis cows (C).

Beta diversity analysis based on Bray–Curtis dissimilarity and visualized using principal coordinate analysis (PCoA) demonstrated significant differences in microbial community composition among groups (PERMANOVA, R² = 0.25, P = 0.0322), explaining 26.01% and 18.85% of variation along axis 1 and axis 2, respectively (**Figure 4**). Pairwise analysis further showed distinct clustering between healthy heifers and cervicitis cows (R² = 0.22, P = 0.0248), with 27.8% and 21.94% variation explained by axis 1 and axis 2, respectively (**Figure 5**).

**Figure 4:**
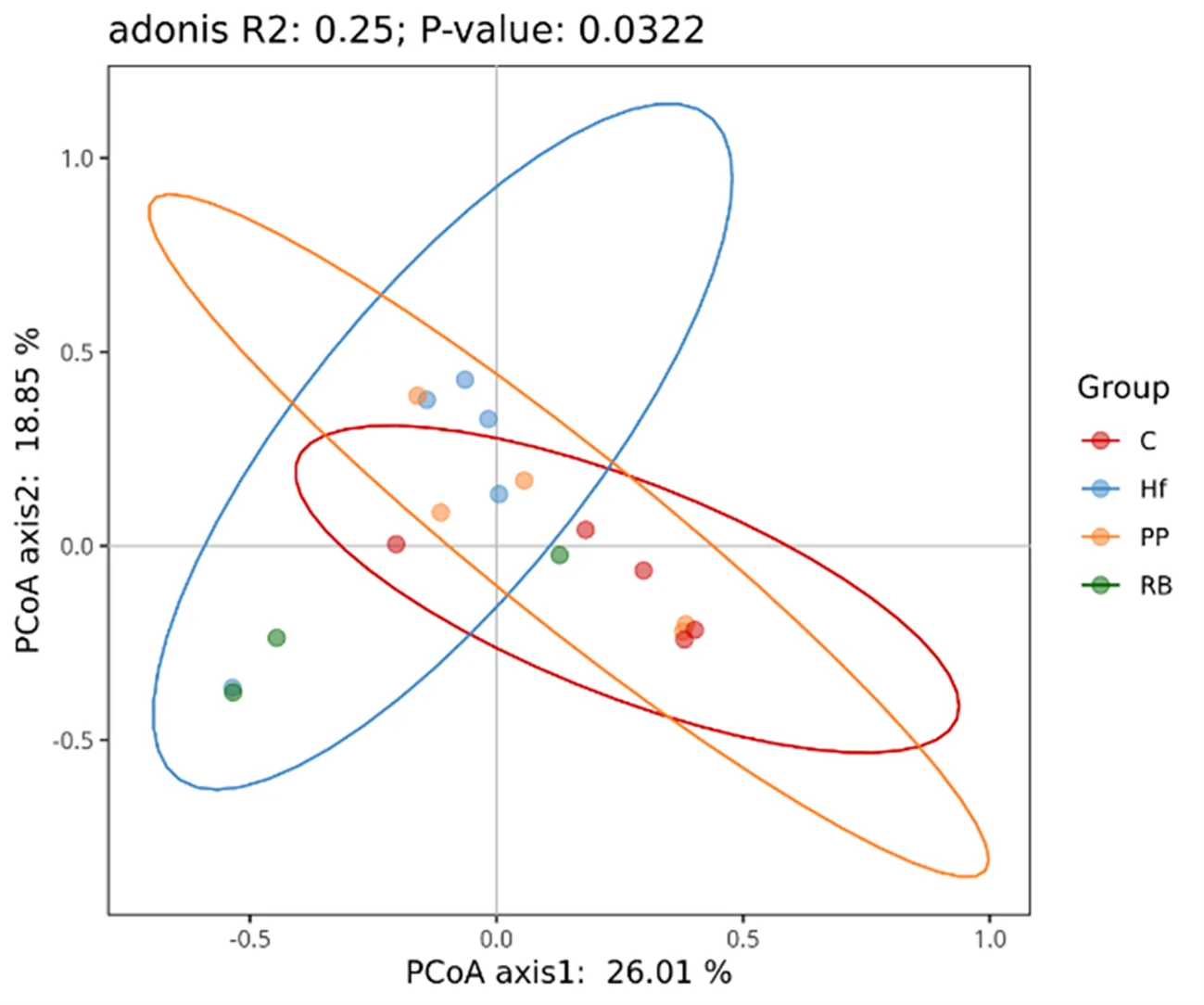
Principal coordinate analysis plot (PCoA) of the Bray-Curtis dissimilarity index representing the comparison among the groups. C, Cervicitis; Hf, Healthy heifers; RB, Repeat breeding; PP, Cows within 35 days postpartum period.

**Figure 5:**
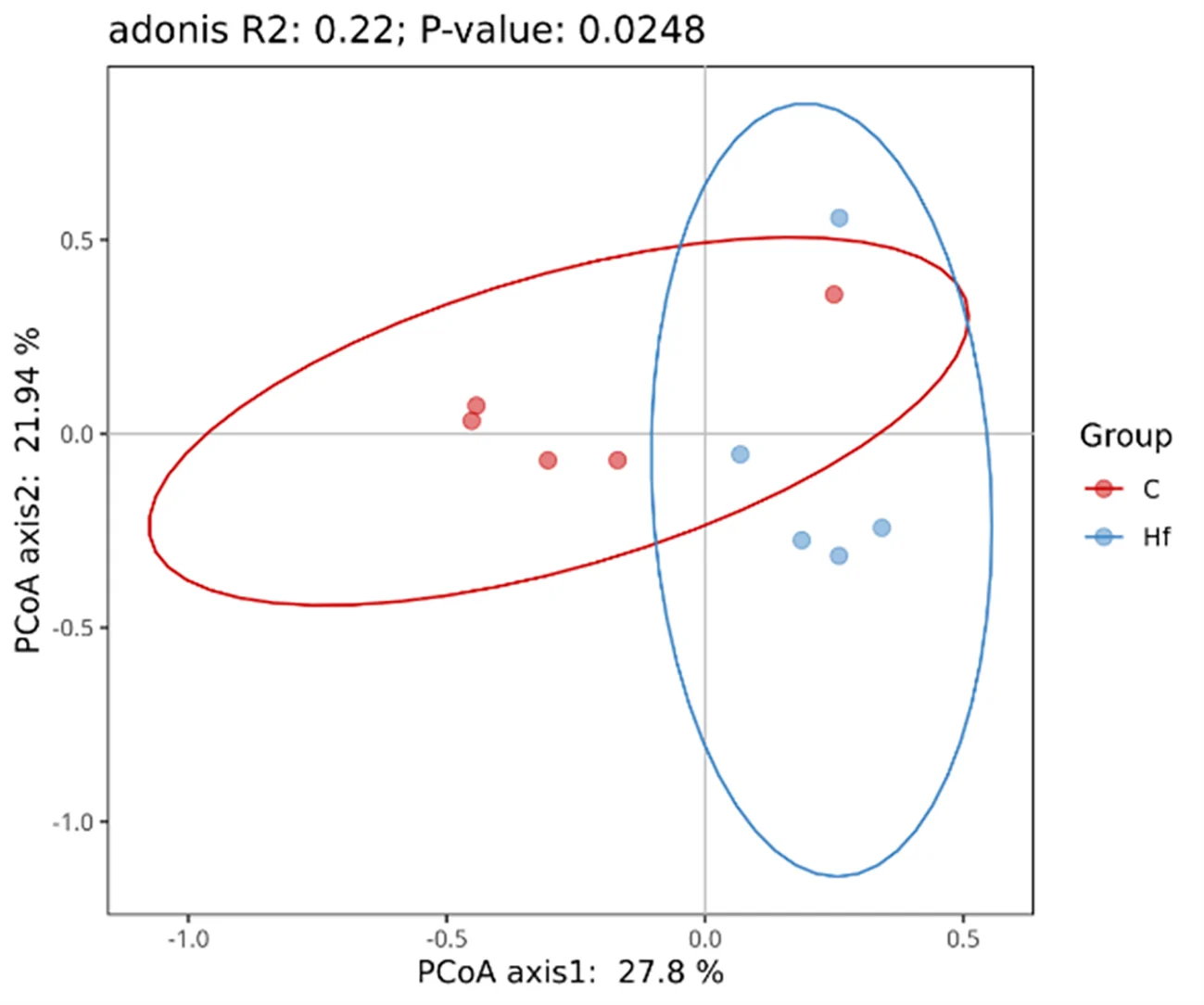
Principal coordinate analysis plot (PCoA) of the Bray-Curtis dissimilarity index representing the comparison between healthy heifers (Hf) and cows with cervicitis (C).

### 4.6 Taxonomic composition of microbial communities

The relative abundance of bacterial phyla varied among study groups (**Figure 6**). In healthy heifers, Bacillota (61.8%) dominated, followed by Pseudomonadota (22.4%), Actinomycetota (8.0%), and Mycoplasmatota (1.4%). In cows within 35 days postpartum, Bacillota (28.5%) was predominant, followed by Pseudomonadota (23.9%), Fusobacteriota (22.4%), Actinomycetota (12.4%), Bacteroidota (8.3%), and Mycoplasmatota (3.2%). Cows with cervicitis showed a distinct profile dominated by Pseudomonadota (36.3%) and Fusobacteriota (30.8%), followed by Bacillota (12.7%), Bacteroidota (10.2%), Actinomycetota (7.6%), and Cyanobacteriota (1.6%). In repeat breeding cows, Bacillota (46.3%) was predominant, followed by Pseudomonadota (28.5%) and Mycoplasmatota (16.0%).

**Figure 6:**
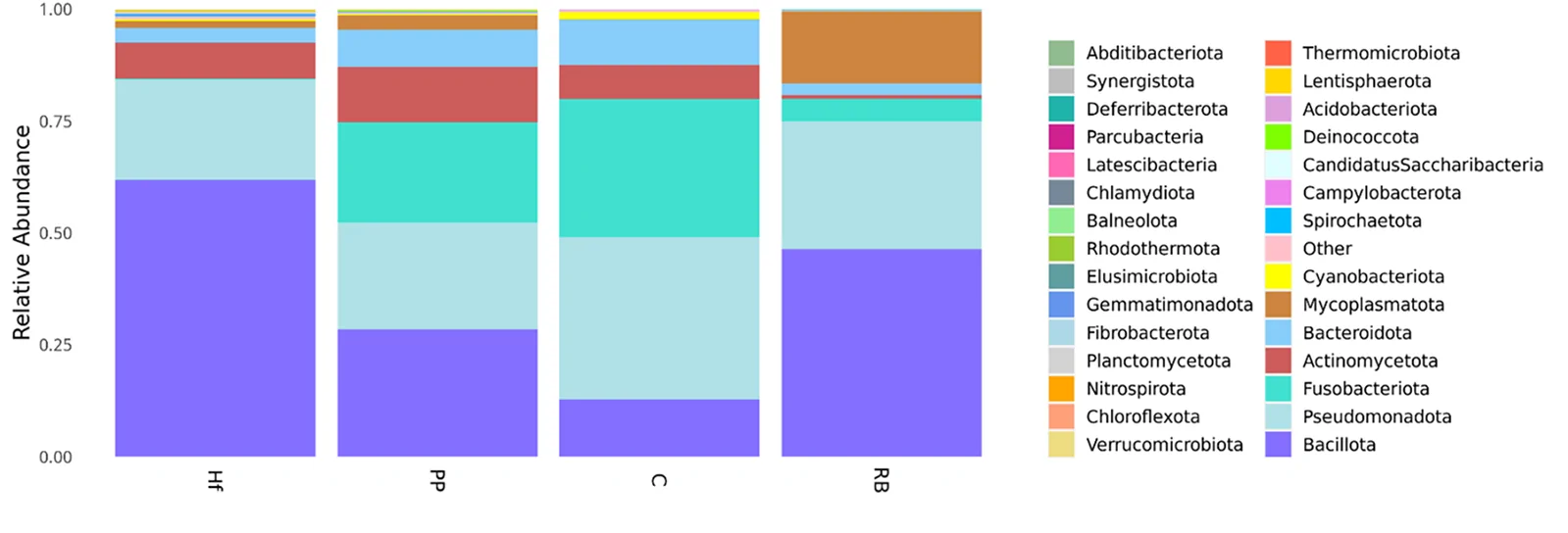
Relative abundance of bacteria across the groups at the phylum level. C, Cervicitis; Hf, Healthy heifers; RB, Repeat breeding; PP, Cows within 35 days postpartum period.

Overall, Bacillota (37.3%) was the most abundant phylum across all samples, followed by Pseudomonadota (27.8%), Fusobacteriota (14.6%), Actinomycetota (7.2%), Bacteroidota (6.1%), and Mycoplasmatota (5.2%), collectively accounting for 98.3% of total sequences. Fusobacteria were absent in healthy heifers but present in other groups, suggesting a potential association with altered reproductive status.

### 4.7 Identification of differentially abundant taxa

LEfSe analysis identified distinct group-specific enriched taxa (**Figure 7**). In repeat-breeding cows, Synergistales (LDA = 2.8) and Filifactor (LDA = 2.2) were significantly enriched. Cows with cervicitis were characterized by enrichment of Weeksellaceae (LDA = 4.0) and Chryseobacterium (LDA = 3.0). Healthy heifers showed the highest diversity of enriched taxa, including Histophilus (LDA = 3.5), Campylobacteria (LDA = 3.4), Nocardioidaceae (LDA = 3.2), Cellulomonadaceae (LDA = 2.3), Selenomonas (LDA = 2.2), Leptotrichiaceae (LDA = 2.2), Leptotrichia (LDA = 2.2), and Actinotalea (LDA = 2.1).

**Figure 7:**
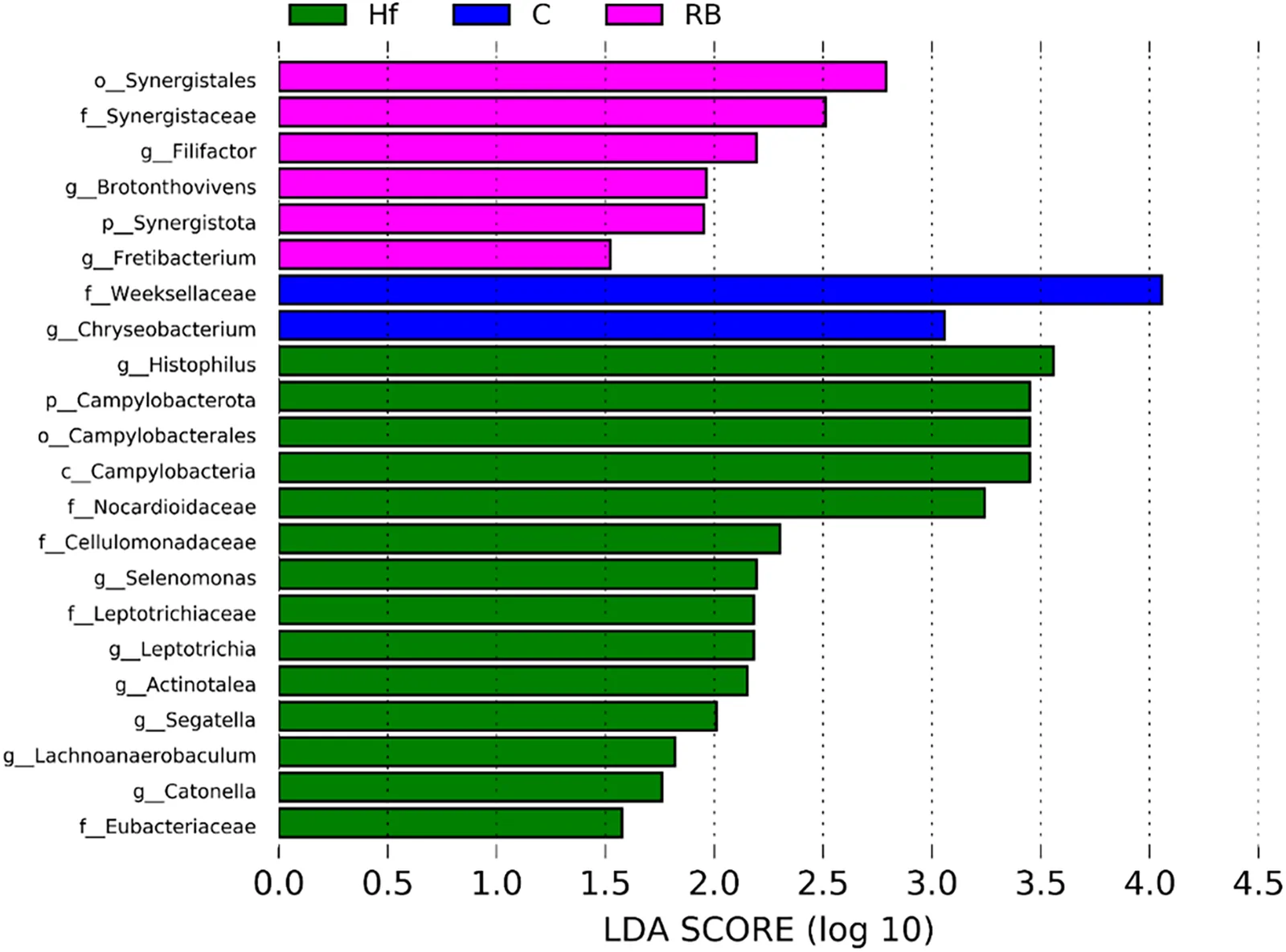
Enriched taxa associated with specific reproductive conditions. Colors of bars represent different groups, and the lengths of bars indicate linear discriminant analysis score (LDA) (LDA scores> 2 are considered enriched taxa). Hf, Healthy heifers; C, Cervicitis; RB, Repeat breeding; PP, Cows within 35 days postpartum period.

### 4.8 Pairwise comparison of differentially abundant bacterial taxa among groups

In cows with cervicitis, Chryseobacterium, Fusobacterium (LDA = 5.2), and Porphyromonas (LDA = 4.5), along with the phyla Fusobacteriota (LDA = 5.2) and Bacteroidota (LDA = 4.6), were significantly enriched compared to healthy heifers. In repeat-breeding cows, Filifactor, along with Synergistota (LDA = 2.7) and Fretibacterium (LDA = 2.1), showed higher abundance than in healthy heifers. In contrast, healthy heifers exhibited enrichment of several taxa, including the phyla Bacillota, Campylobacteriota, and Spirochaetota, and genera such as Campylobacter, Trueperella, and Histophilus, compared with cows with cervicitis and repeat breeding (**Figure 8**).

**Figure 8:**
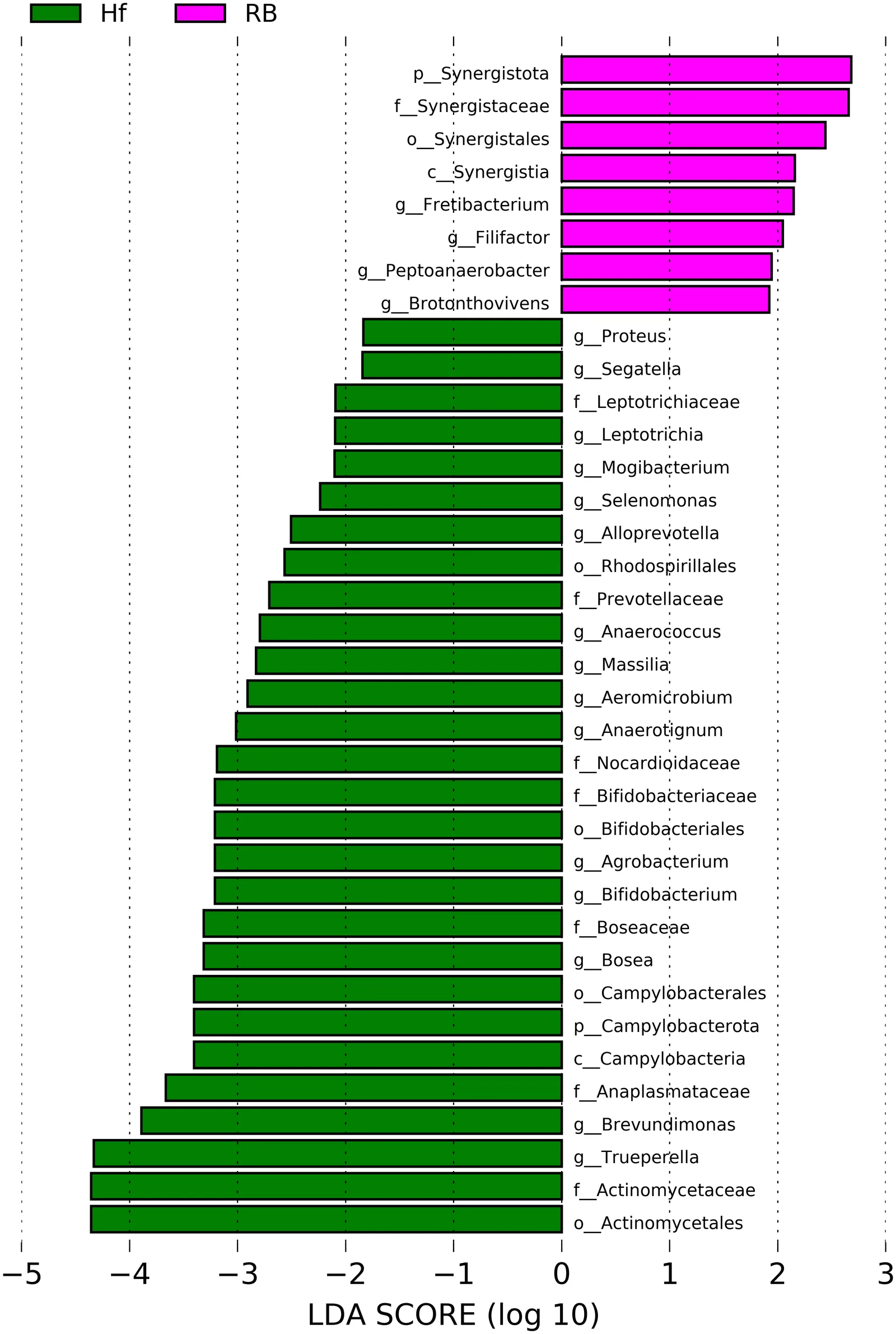
Enriched taxa associated with Hf and RB (left), and Hf and C (right). Colors of bars represent different groups, and the lengths of bars indicate linear discriminant analysis scores (LDA) (LDA *scores*> 2 are considered enriched taxa). Hf, Healthy heifers; RB, Repeat breeding.

### 4.9 Correlation of the bacteria at the species level

A strong positive correlation was observed between *Porphyromonas levii* and *Fusobacterium necrophorum* (rs = 0.96), and between *Trueperella pyogenes* and *Proteus mirabilis* (rs = 0.86). In contrast, moderate negative correlations were detected between *T. pyogenes* and *F. necrophorum* (rs = −0.5), *T. pyogenes* and *P. levii* (rs = −0.4), *Priestia megaterium* and *Helcococcus ovis* (rs = −0.5), and *Bacillus pumilus* and *T. pyogenes* (rs = −0.4) (**Figure 9**).

**Figure 9:**
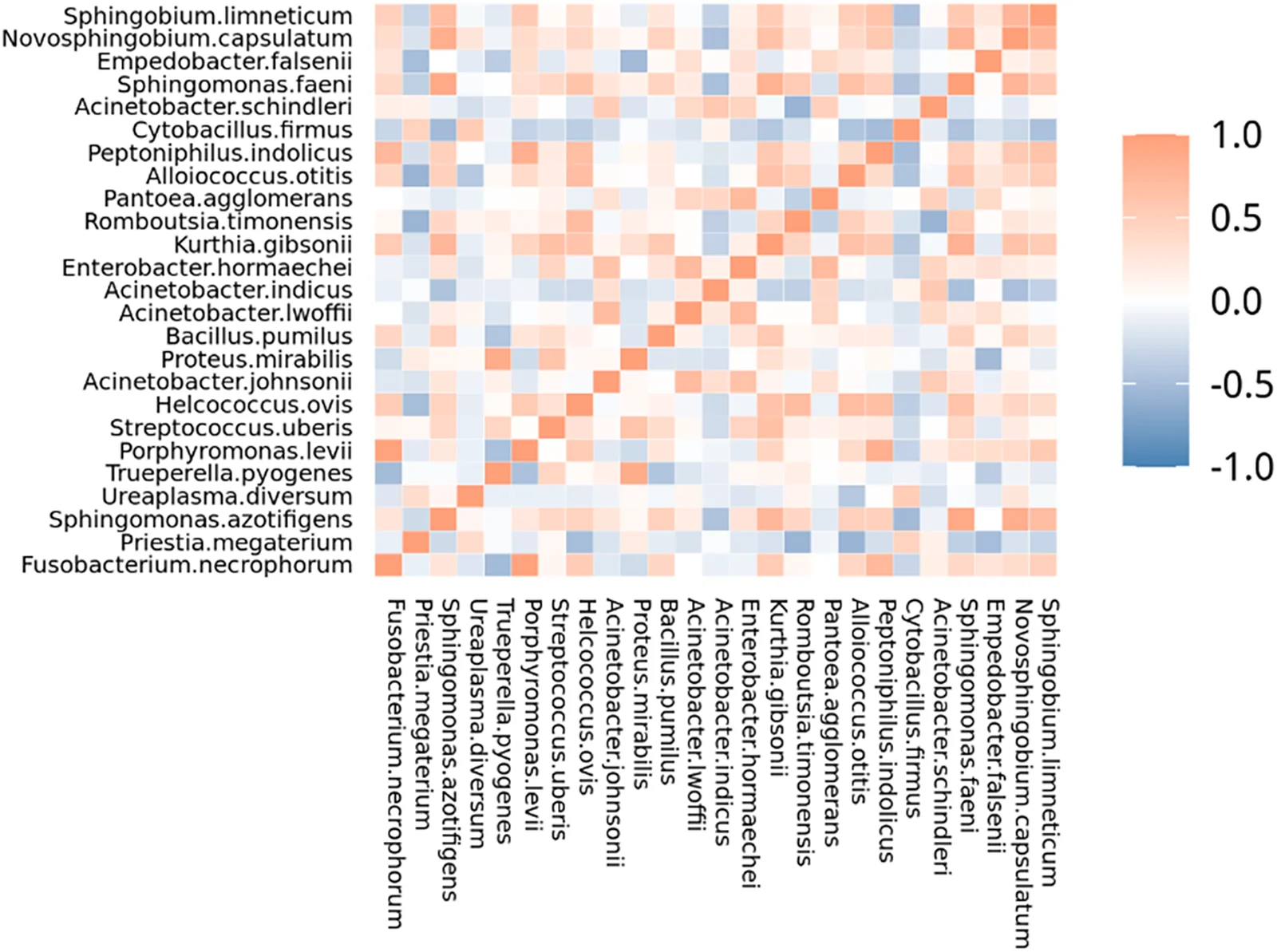
Correlation between the species level based on abundance (relative abundance > 0.5%). The index (-1.0 to +1.0) shows the correlation coefficient value. A correlation coefficient (r_s_) less than 0.2 is not included in the heat map.

## 5 Discussion

The importance of vaginal microbiota in ensuring successful pregnancy in cattle, humans, and other mammals has been well documented (Rodrigues et al., 2015; Quereda et al., 2020; Amat et al., 2021; Moore et al., 2023; Brulin et al., 2024). However, comparative information on vaginal microbiota between healthy heifers and cattle with impaired reproductive performance, such as repeat breeding and cervicitis, remains limited. To our knowledge, only one study has compared the vaginal microbiota of healthy heifers and repeat-breeding cows (Moreno et al., 2022). Therefore, this study expands current knowledge by investigating the cervico-vaginal microbiota of healthy heifers and cows with impaired reproductive performance, including cervicitis, for the first time.

### 5.1 Bacteria isolates from the external os of the cervix using the microbiological culture method and their antimicrobial resistance

In this study, *Enterobacter* spp., *Micrococcus* spp., *Acinetobacter* spp., *Bacillus* spp., *Staphylococcus* spp., *Salmonella* spp., *Escherichia coli*, *Pseudomonas* spp., *Shigella* spp., and *Citrobacter* spp. were isolated from the external os of the cervix. These bacteria have previously been categorized based on their pathogenic potential in the uterus, with *E. coli* recognized as a primary pathogen, Acinetobacter, *Bacillus*, and *Staphylococcus* spp. as potential pathogens, and *Enterobacter* and *Micrococcus* spp. as opportunistic contaminants (Sheldon et al., 2006). Similar isolates have been reported from vaginal discharge of subclinical endometritis cases in Nepal (Panta and Prasad Mandal, 2024) and uterine cultures in Slovakia (Ballas et al., 2021). Likewise, *Micrococcus* and *Staphylococcus* spp. have been isolated from postpartum anestrus and silent heat cows in England (Williams et al., 2005).

Although many of these bacteria are not primary pathogens, they can become clinically relevant under conditions of dysbiosis, where microbial imbalance compromises host reproductive defenses (Williams et al., 2007). Such dysbiosis may result from altered metabolism, immune suppression, antibiotic exposure, or changes in host health status (Adnane and Chapwanya, 2024; Dias et al., 2024). Reproductive tract infections are increasingly considered to arise from polymicrobial interactions rather than single pathogenic agents (LeBlanc et al., 2011). The early postpartum period is particularly critical, as uterine involution and microbial clearance determine subsequent fertility (López-Helguera et al., 2016). In the present study, a single *E. coli* isolate was recovered from cows within 35 days postpartum, suggesting progression toward clearance of postpartum contamination. However, this should be interpreted cautiously, as bacterial identification was based on morphology, Gram staining, hemolysis, and biochemical tests. Furthermore, the lack of anaerobic culture may have limited the detection of anaerobic bacteria. Future studies should incorporate molecular identification methods and anaerobic culture to improve bacterial characterization.

The antibiotic susceptibility profile revealed a high prevalence of MDR isolates and elevated MAR indices across bacterial genera, indicating possible exposure to high antimicrobial pressure in the environment (Bhattarai et al., 2024). Notably, a high proportion of Salmonella spp. were resistant to erythromycin, consistent with reports from South Asia and global studies describing resistance among Enterobacteriaceae in livestock (Harun et al., 2024; Pires et al., 2024). Complete resistance of *Salmonella* spp. to ciprofloxacin observed in this study aligns with global concerns, which are attributed to outer membrane barriers, efflux pumps, and antibiotic-inactivating enzymes (Pires et al., 2024). In contrast, MDR-negative Salmonella isolates have been reported in cattle uterine discharges in Ethiopia, indicating geographical variation in resistance patterns (Mekibib et al., 2024).

Members of the *Enterobacter, Staphylococcus, and Acinetobacter* genera belong to the ESKAPE group of nosocomial pathogens, known for their multidrug resistance and clinical importance in human infections (Gupta et al., 2024; Chong et al., 2025; Sah et al., 2025). These organisms are classified by the World Health Organization as critical, high, and medium priority pathogens due to their public health significance (WHO, 2024). In the present study, a high proportion of ESKAPE-related isolates were MDR-positive, exceeding rates reported in clinical human isolates from hospitals in Nepal (Basnet et al., 2024; Sah et al., 2025). Similar findings have been reported in livestock, including MDR *Staphylococcus* spp. from cattle and buffalo milk in Nepal (Shrestha et al., 2021; Chowdhury et al., 2025) and aborted cattle in Russia (Zaitsev et al., 2022), supporting the persistence of resistant bacteria in animal production systems.

Overall, these findings may indicate that MDR ESKAPE pathogens are not confined to clinical settings but are also present in livestock environments, posing a potential zoonotic and occupational health risk for farmers, veterinarians, and food handlers.

### 5.2 Composition of vaginal bacterial communities using 16S rRNA sequencing

Several studies have demonstrated that the bovine reproductive tract microbiota varies with reproductive status and may be associated with pregnancy outcomes in both cattle and humans (Moreno et al., 2022; Brulin et al., 2024). In agreement with these reports, the present study showed clear differences in vaginal bacterial composition among reproductive groups. Healthy heifers exhibited higher microbial richness and diversity compared to cows with cervicitis, suggesting a positive association between microbial diversity and reproductive health, consistent with previous findings (Moreno et al., 2022; Dias et al., 2024). Reduced OTU abundance and enrichment of potentially pathogenic taxa in cervicitis cases further indicate dysbiosis associated with reproductive disease (LeBlanc et al., 2011; Laguardia-Nascimento et al., 2015).

Across all groups, Bacillota, Pseudomonadota, Actinomycetota, and Mycoplasmatota were consistently detected, suggesting a core vaginal microbiota in cattle, in agreement with previous studies (Quereda et al., 2020; Amat et al., 2021; Brulin et al., 2024). These phyla are widely distributed across host-associated environments and play important roles in microbial homeostasis (Clemmons et al., 2017). In contrast, Fusobacteriota and increased Bacteroidota were more prominent in cows with impaired reproductive performance. These taxa have been repeatedly associated with uterine diseases such as metritis and pregnancy failure, supporting their role as indicators of reproductive tract dysbiosis (Rodrigues et al., 2015; Moore et al., 2023).

Although a large proportion of OTUs were shared among groups, indicating a conserved core microbiome, distinct differences in community structure between healthy and diseased animals highlight clear microbial shifts associated with reproductive status. In this study, Fusobacteriota and Bacteroidota were significantly enriched in cervicitis cases, consistent with their association with reproductive tract infections (Paiano et al., 2022; Dias et al., 2024). Conversely, Bacillota was more abundant in healthy heifers, suggesting a potential role in maintaining vaginal homeostasis (Miranda-CasoLuengo et al., 2019). Repeat-breeding cows were dominated by Bacillota and Pseudomonadota, consistent with previous reports (Moreno et al., 2022), although no strong enrichment of classical pathogens was observed, indicating a dysbiotic but less pathogen-dominant state.

Notably, *Fusobacterium necrophorum* and *Porphyromonas levii* were significantly enriched in cervicitis cases compared to healthy heifers and postpartum cows. *F. necrophorum* is a well-established reproductive pathogen, while *P. levii* is an emerging pathogen associated with uterine infections (Galvão et al., 2019; Adnane and Chapwanya, 2024). The postpartum period is critical for uterine involution and microbial clearance, and failure to eliminate such pathogens may predispose cows to reproductive disorders (Sheldon et al., 2008). In addition, *Chryseobacterium* (Weeksellaceae) was enriched in cattle with cervicitis. Although its role in cattle is not well understood, it has been detected in human reproductive disorders and cancer cases, suggesting possible pathogenic potential (Moreno et al., 2022; Gao et al., 2024).

In repeat-breeding cows, *Filifactor* and Synergistota were significantly enriched, while typical pathogenic taxa were not strongly dominant. *Filifactor* has been associated with uterine infections in cattle and may contribute to reproductive dysfunction, although its functional role remains unclear (Rashid et al., 2025). The reduced presence of beneficial taxa and altered microbial composition suggest dysbiosis as a possible underlying factor in repeat breeding.

Healthy heifers showed the highest microbial diversity and enrichment of several commensal or potentially beneficial taxa, including *Leptotrichia* and *Bifidobacterium pseudolongum*. Higher microbial diversity is generally associated with resilience and maintenance of reproductive tract homeostasis (Dias et al., 2024). *B. pseudolongum* is considered a beneficial bacterium with probiotic potential and GRAS status, while *Leptotrichia* has been reported in healthy reproductive tracts of both cattle and humans (Arsène et al., 2021; Patel et al., 2022). Interestingly, some taxa traditionally considered pathogenic, such as *Histophilus, Trueperella*, and *Campylobacter*, were also detected in healthy heifers, consistent with previous studies (Karstrup et al., 2017; Brulin et al., 2024). Their presence likely reflects a commensal state under host immune control rather than active infection, as disease expression depends on host immunity, environmental stress, and bacterial virulence factors (Sheldon et al., 2008; Bicalho et al., 2016).

Overall, these findings suggest that reproductive status in cattle is associated with differences in vaginal microbial composition, with healthy heifers generally exhibiting more stable and diverse microbial communities than cows with reproductive disorders. However, these findings should be interpreted with caution, as the study was exploratory in nature and the animal groups were not fully matched for potentially confounding factors, including age, parity, lactation status, postpartum stage, farm, and management practices. In addition, some replacement samples were collected from different locations due to poor DNA quality in the original samples, which may have introduced location-related variation. Consequently, the observed microbial differences cannot be attributed solely to disease status but rather represent associations that warrant confirmation in larger, well-controlled longitudinal studies.

### 5.3 Relationships among major pathogenic bacteria present in the vagina

In this study, a positive correlation was observed between *Fusobacterium necrophorum* and *Porphyromonas levii*, while both showed a negative correlation with *Trueperella pyogenes*. Although previous studies have reported co-occurrence of these bacteria and suggested potential synergism in maintaining an anaerobic environment (Adnane and Chapwanya, 2024; Gupta et al., 2024), our findings only partially support this hypothesis, confirming a stronger association between *F. necrophorum* and *P. levii*. However, these correlations should be interpreted with caution because of the exploratory nature of this study, the relatively small sample size, and the potential influence of confounding factors, including difference in age, parity, postpartum stage, farm and management practices among the study groups. Therefore, the observation association should be considered hypothesis-generating rather than evidence of direct ecological interactions, and further investigation using larger, well-controlled cohorts is required to clarify their ecological interactions.

A positive correlation was also observed between *T. pyogenes* and *Proteus mirabilis*, consistent with previous reports of their co-occurrence in uterine infections, although the underlying mechanism remains unclear (Williams et al., 2005; Dolezel et al., 2010). In contrast, a negative correlation was detected between *Priestia megaterium* and *Helcococcus ovis*. *H. ovis* is an emerging reproductive pathogen associated with metritis and abortion in cattle (Cunha et al., 2024; Gao et al., 2024), whereas *P. megaterium* is widely recognized for its probiotic potential and antimicrobial activity against several pathogenic bacteria. This inverse relationship suggests a possible inhibitory effect of *P. megaterium* on *H. ovis*, which warrants further validation. Similarly, a negative correlation between *Bacillus pumilus* and *T. pyogenes* was observed. While *B. pumilus* has been reported to exhibit antimicrobial activity against enteric pathogens such as *E. coli* and *Salmonella*, it has also been associated with reproductive tract infections in some studies, indicating a dual functional role depending on environmental context (Elshaghabee et al., 2017; Ballas et al., 2021). Nevertheless, correlation analyses cannot establish causality, and the observed relationships may also reflect indirect association driven by host or environmental factors rather than direct microbial interactions.

Although a proportion of bacterial isolates (11.4%) remained unidentified due to methodological limitations, the overall findings provide important insights into microbial interactions within the bovine reproductive tract. Correlation analysis further suggests potential ecological relationships among pathogenic and commensal bacteria. While these associations require validation in larger cohorts, the present study provides a foundational framework for understanding microbial interactions that may influence reproductive health and fertility outcomes.

### 5.4 Study limitations

This study has several limitations that should be considered when interpreting the findings. First, the study was conducted using a relatively small and uneven sample size across groups, primarily due to the limited availability of eligible animals under field conditions. Second, convenience sampling from both farmer-owned and institutional farms may have introduced selection bias and resulted in variability in animal-related factors such as age, parity, lactation status, nutrition, and management practices. Third, the cross-sectional study design does not allow assessment of temporal dynamics of the cervico-vaginal microbiota or causal relationships between microbial composition and reproductive disorders. In addition, bacterial culture-based identification relied on biochemical methods without molecular confirmation (e.g., MALDI-TOF or sequencing), which may limit taxonomic resolution. For the microbiome analysis, no longitudinal sampling or follow-up of animals was performed, and potential farm- or environment-associated effects could not be fully controlled. Finally, differential abundance and correlation analyses were conducted on a limited number of samples, and therefore findings such as LEfSe results and species-level correlations should be interpreted as exploratory and hypothesis-generating rather than confirmatory.

In conclusion, this study provides preliminary evidence of distinct cervico-vaginal microbial profiles associated with reproductive status in cattle. Dysbiosis characterized by reduced microbial diversity and enrichment of pathogenic taxa such as *Fusobacterium necrophorum*, *Porphyromonas levii*, *Chryseobacterium*, and *Filifactor* was observed in cows with reproductive disorders. In contrast, healthy heifers exhibited higher microbial diversity and greater relative abundance of taxa such as *Leptotrichia* and *Bifidobacterium pseudolongum*, suggesting a more balanced microbial community. A high prevalence of multidrug-resistant bacteria, including ESKAPE-associated organisms, highlights the presence of antimicrobial resistance within the bovine reproductive tract. Additionally, observed bacterial correlations suggest potential microbial interactions that may be associated with reproductive status. However, given the cross-sectional design and limited sample size, these findings should be interpreted as exploratory and hypothesis-generating. Further large-scale, longitudinal studies are required to validate these associations and elucidate their biological significance.

## Data Availability

The original contributions presented in the study are publicly available. This data can be found here: NCBI Sequence Read Archive, accession PRJNA1529812.
