# Supplementary material for: Cervico-vaginal microbiota and antimicrobial resistance patterns in relation to different reproductive statuses in cattle: a field study from Nepal
Source: Front Microbiomes. 2026 Oct 5;5:1900716. doi: 10.3389/frmbi.2026.1900716 (PMC13677908; doi:10.3389/frmbi.2026.1900716)
Supplement: Supplementary file 1 [file DataSheet1.pdf]

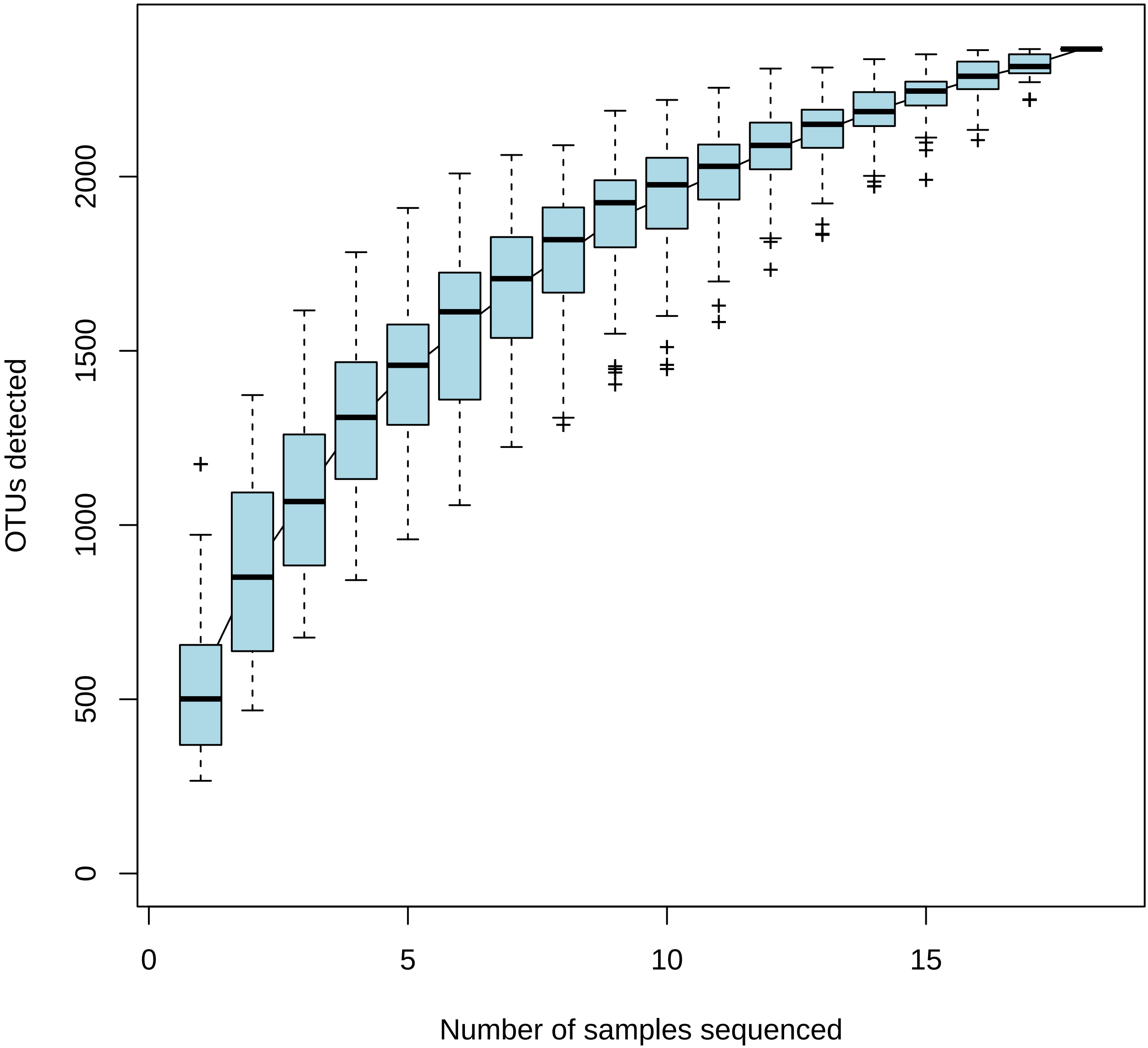
OTUs detected
0
500
1000
1500
2000
0
5
10
15
Number of samples sequenced
